# Supplementary material for: Performance of some early-maturing groundnut (Arachis hypogaea L.) genotypes and selection of high-yielding genotypes in the potato-fallow system
Source: PLoS One. 2023 Apr 25;18(4):e0282438. doi: 10.1371/journal.pone.0282438 (PMC10128977; doi:10.1371/journal.pone.0282438)
Supplement: S1 Table — (DOCX) [file pone.0282438.s001.docx]

S1 Table: Performance of genotypes for maturity, SCMR and yield related characters at Junagadh over three seasons

| Genotype | Year | DFI (days) | DFF (days) | DM (days) | SCMR | SLA | PYLP (g) | HPW (g) | SP (%) | KL (mm) | KW (mm) | KLWR |
| --- | --- | --- | --- | --- | --- | --- | --- | --- | --- | --- | --- | --- |
| Chico | 2017 | 23.67 | 26.67 | 95.33 | 24.47 | 221.53 | 6.51 | 61.99 | 75.10 | 12.90 | 6.97 | 1.85 |
|  | 2018 | 26.00 | 28.33 | 98.33 | 33.17 | 249.38 | 7.40 | 75.22 | 76.79 | 13.47 | 7.07 | 1.91 |
|  | 2019 | 22.67 | 25.67 | 96.00 | 20.87 | 350.33 | 7.54 | 48.94 | 59.56 | 5.77 | 3.23 | 1.79 |
| NRCG CS 254 | 2017 | 23.33 | 26.00 | 95.33 | 31.03 | 201.51 | 14.15 | 83.46 | 70.78 | 13.67 | 7.53 | 1.81 |
|  | 2018 | 23.33 | 26.33 | 100.00 | 37.10 | 248.60 | 7.51 | 89.89 | 73.43 | 13.67 | 8.00 | 1.71 |
|  | 2019 | 23.67 | 26.33 | 96.00 | 25.80 | 294.10 | 16.27 | 73.62 | 59.62 | 7.07 | 3.43 | 2.06 |
| NRCG CS 292 | 2017 | 22.67 | 26.33 | 98.00 | 27.97 | 199.65 | 6.24 | 69.12 | 72.24 | 12.40 | 7.23 | 1.72 |
|  | 2018 | 25.00 | 27.33 | 99.00 | 33.70 | 241.62 | 9.06 | 84.73 | 74.81 | 14.87 | 8.10 | 1.86 |
|  | 2019 | 23.67 | 25.67 | 96.67 | 21.37 | 340.94 | 6.54 | 60.80 | 60.95 | 5.93 | 3.30 | 1.80 |
| NRCG CS 313 | 2017 | 22.33 | 25.00 | 96.67 | 30.17 | 182.86 | 9.52 | 75.98 | 72.22 | 13.90 | 8.23 | 1.69 |
|  | 2018 | 23.33 | 26.33 | 100.00 | 33.57 | 195.45 | 5.50 | 88.94 | 72.70 | 14.37 | 8.33 | 1.73 |
|  | 2019 | 24.33 | 27.33 | 97.67 | 21.77 | 371.61 | 7.33 | 59.68 | 59.78 | 6.30 | 3.83 | 1.65 |
| NRCG CS 330 | 2017 | 22.00 | 24.00 | 96.33 | 19.53 | 257.76 | 5.83 | 59.63 | 68.40 | 11.40 | 7.27 | 1.57 |
|  | 2018 | 23.67 | 27.67 | 98.67 | 28.13 | 261.49 | 4.90 | 64.33 | 72.27 | 11.77 | 7.80 | 1.51 |
|  | 2019 | 23.00 | 25.00 | 94.67 | 17.80 | 303.54 | 4.05 | 51.41 | 60.12 | 5.67 | 3.13 | 1.82 |
| NRCG CS 40 | 2017 | 22.00 | 25.00 | 97.33 | 30.40 | 213.93 | 3.69 | 72.67 | 62.35 | 13.73 | 7.77 | 1.77 |
|  | 2018 | 24.00 | 27.67 | 105.67 | 35.60 | 247.53 | 5.72 | 89.05 | 63.93 | 14.63 | 7.57 | 1.94 |
|  | 2019 | 23.67 | 27.67 | 99.00 | 20.63 | 294.14 | 7.52 | 72.96 | 48.43 | 6.80 | 3.60 | 1.89 |
| NRCG CS 404 | 2017 | 22.67 | 25.33 | 96.33 | 27.90 | 209.99 | 5.25 | 53.65 | 74.75 | 11.00 | 7.47 | 1.48 |
|  | 2018 | 24.33 | 26.67 | 98.33 | 34.67 | 251.67 | 4.63 | 62.97 | 75.67 | 11.80 | 7.63 | 1.54 |
|  | 2019 | 23.33 | 26.67 | 96.33 | 25.10 | 352.51 | 5.80 | 48.87 | 63.81 | 5.50 | 3.60 | 1.54 |
| NRCG CS 445 | 2017 | 21.67 | 24.33 | 96.67 | 31.27 | 178.76 | 5.79 | 77.04 | 66.23 | 14.40 | 8.40 | 1.72 |
|  | 2018 | 26.33 | 28.67 | 99.33 | 32.80 | 253.01 | 7.81 | 87.63 | 69.89 | 15.60 | 8.97 | 1.74 |
|  | 2019 | 23.00 | 27.00 | 99.00 | 26.83 | 267.10 | 6.72 | 71.00 | 57.16 | 6.50 | 3.53 | 1.84 |
| NRCG CS 446 | 2017 | 20.67 | 22.33 | 97.00 | 30.27 | 181.51 | 8.54 | 76.90 | 65.72 | 16.20 | 8.13 | 1.99 |
|  | 2018 | 25.33 | 27.33 | 104.00 | 36.57 | 236.44 | 7.35 | 103.77 | 68.13 | 16.67 | 8.30 | 2.01 |
|  | 2019 | 23.67 | 28.00 | 100.00 | 24.50 | 293.91 | 9.23 | 81.64 | 49.34 | 7.93 | 3.57 | 2.23 |
| NRCG CS 62 | 2017 | 20.00 | 22.00 | 96.00 | 30.57 | 211.83 | 5.24 | 55.35 | 70.38 | 11.73 | 5.70 | 2.23 |
|  | 2018 | 23.67 | 26.00 | 101.00 | 33.17 | 249.41 | 3.93 | 55.76 | 71.80 | 11.80 | 6.90 | 1.71 |
|  | 2019 | 22.67 | 26.67 | 95.67 | 21.70 | 307.44 | 4.15 | 59.29 | 46.65 | 5.77 | 3.17 | 1.84 |
| PM 1 | 2017 | 24.67 | 28.33 | 98.00 | 31.37 | 175.98 | 7.00 | 68.71 | 68.48 | 12.87 | 7.57 | 1.70 |
|  | 2018 | 28.00 | 30.33 | 106.00 | 36.00 | 221.04 | 4.82 | 73.55 | 66.74 | 14.27 | 7.70 | 1.86 |
|  | 2019 | 23.00 | 25.67 | 95.33 | 24.63 | 314.06 | 6.85 | 54.08 | 59.60 | 5.93 | 3.70 | 1.61 |
| TAG 24 | 2017 | 22.33 | 24.33 | 95.33 | 29.30 | 205.97 | 6.84 | 90.07 | 71.81 | 13.43 | 7.97 | 1.69 |
|  | 2018 | 25.67 | 28.00 | 97.33 | 32.47 | 264.29 | 6.50 | 94.42 | 73.26 | 13.80 | 8.93 | 1.55 |
|  | 2019 | 23.67 | 26.33 | 97.33 | 21.87 | 266.98 | 7.42 | 69.52 | 61.68 | 6.01 | 3.67 | 1.65 |
