# Supplementary material for: Performance of some early-maturing groundnut (Arachis hypogaea L.) genotypes and selection of high-yielding genotypes in the potato-fallow system
Source: PLoS One. 2023 Apr 25;18(4):e0282438. doi: 10.1371/journal.pone.0282438 (PMC10128977; doi:10.1371/journal.pone.0282438)
Supplement: Attachment — Submitted filename: PONE-D-22-26579 - Comments.doc [file pone.0282438.s002.doc]

**PLOS ONE**

**Performance of some early-maturing groundnut genotypes and selection of high-yielding genotypes in the potato-fallow system**

|  | --Manuscript Draft-- |
| --- | --- |
|  |  |
| **Manuscript Number:** | PONE-D-22-26579 |
| **Article Type:** | Research Article |
|  |  |
| **Full Title:** | Performance of some early-maturing groundnut genotypes and selection of high- |
|  | yielding genotypes in the potato-fallow system |
| **Short Title:** | Groundnut in potato fallow |
|  |  |
| **Corresponding Author:** | Sandip K Bera, Ph.D. |
|  | Directorate of Groundnut Research |
|  | Junagadh, Gujarat INDIA |
| **Keywords:** | Potato-fallow, Interspecific derivatives, Pod yield, Shelling percentage |
|  |  |
| **Abstract:** | India is the largest importer of edible oils as it has to meet domestic demand, which |
|  | surpasses its domestic production. To reduce the import of edible oils, there is a scope |
|  | for horizontally expanding groundnut production, especially in the potato-paddy rice- |
|  | fallow system, which requires trait-specific varieties suitable for specific cropping |
|  | systems. Currently, 99% of oilseeds are cultivated in their traditional areas, while only |
|  | 1% of cultivation is done in non-traditional areas. To increase the production of |
|  | oilseeds, both the traditional and non-traditional areas have to be expanded by around |
|  | 5 million ha according to the strategies of Oilseeds and oil palm by 2020–26 to achieve |
|  | self-reliance through a campaign called Atmanirbhar Bharat launched by the |
|  | Government of India (NFSM-Oilseeds and Oil palm). Non-traditional areas are all |
|  | expanded along river beds, particularly in paddy rice-potato-fallow in the North Eastern |
|  | States, to utilize residual soil moisture. To assess performance and adaptability, nine |
|  | interspecific derivatives of groundnut have been evaluated in the potato-fallow system |
|  | in Deesa in Gujarat and Mohanpura in West Bengal and non-potato and fallow lands in |
|  | Junagadh during Kharif 2020. The combined analysis of variance (ANOVA) showed |
|  | significant effects of genotype-by-environment (G×E) interaction on pod yield and its |
|  | Component traits. Results of “mean vs. stability” identified the interspecific derivative |
|  | NRCGCS 446 and the variety TAG 24 as the most stable genotypes with the most |
|  | useful traits. Genotype GG 7 performed better for pod yield in the Junagadh, whereas |
|  | NRCGCS 254 performed better in terms of pod yield in Mohanpur. Low heritability |
|  | estimates and significant G×E interaction found for the days to flowering indicated |
|  | complex inheritance and influence of environment on their expression. The shelling |
|  | Percentage had a significant negative correlation with days to 50% flowering, days to |
|  | maturity, SCMR, HPW, and KLWR, suggesting negative associations between |
|  | maturity, component traits, and seed size realization. |
| **Order of Authors:** | Gangadhara K, Ph. D. |
|  |  |
|  | Ajay BC, Ph. D. |
|  |  |
|  | Kona Praveen, Ph. D. |
|  |  |
|  | Kirti Rani, Ph. D. |
|  |  |
|  | Narendra Kumar, Ph. D. |
|  |  |
|  | Sandip K Bera, Ph.D. |
|  |  |
| **Additional Information:** |  |
| **Question** | **Response** |
|  |  |
| **Financial Disclosure** | No |
| Enter a financial disclosure statement that |  |
| describes the sources of funding for the |  |
| work included in this submission. Review |  |
|  |  |

Powered by Editorial Manager® and ProduXion Manager® from Aries Systems Corporation

| the [submission guidelines](http://journals.plos.org/plosone/s/submission-guidelines#loc-financial-disclosure-statement) for detailed | | | | | | | |  |
| --- | --- | --- | --- | --- | --- | --- | --- | --- |
|  |  |  | |  |  |  |  |  |
| requirements. View published research | | | | | | | |  |
| articles from [*PLOS ONE*](http://journals.plos.org/plosone/) for specific | | | | | | | |  |
| examples. | |  | | |  | | |  |
| This statement is required for submission | | | | | | | |  |
| and **will appear in the published article** if | | | | | | | |  |
| the submission is accepted. Please make | | | | | | | |  |
| sure it is accurate. | | | | | | | |  |
|  |  |  | |  |  |  |  |  |
| **Unfunded studies** | | | | | | | |  |
| Enter: *The author(s) received no specific* | | | | | | | |  |
| *funding for this work.* | | | | | | | |  |
| **Funded studies** | | | | | | | |  |
| Enter a statement with the following details: | | | | | | | |  |
| • Initials of the authors who received each | | | | | | | |  |
| award | | | | | | | |  |
| • Grant numbers awarded to each author | | | | | | | |  |
| • The full name of each funder | | | | | | | |  |
| • URL of each funder website | | | | | | | |  |
| • Did the sponsors or funders play any role in | | | | | | | |  |
| the study design, data collection and | | | | | | | |  |
| analysis, decision to publish, or preparation | | | | | | | |  |
| of the manuscript? | | | | | | | |  |
| **• NO** - Include this sentence at the end of | | | | | | | |  |
| your statement: *The funders had no role in* | | | | | | | |  |
| *study design, data collection and analysis,* | | | | | | | |  |
| *decision to publish, or preparation of the* | | | | | | | |  |
| *manuscript.* | | | | | | | |  |
| **• YES** - Specify the role(s) played. | | | | | | | |  |
| * typeset | | | | | | | |  |
| **Competing Interests** | | | | | | | | The authors have declared that no competing interests exist |
| Use the instructions below to enter a | | | | | | | |  |
| competing interest statement for this | | | | | | | |  |
| submission. On behalf of all authors, | | | | | | | |  |
| disclose any [competing interests](http://journals.plos.org/plosone/s/competing-interests) that | | | | | | | |  |
|  | | |  | | | |  |  |
| could be perceived to bias this | | | | | | | |  |
| work—acknowledging all financial support | | | | | | | |  |
| and any other relevant financial or non- | | | | | | | |  |
| financial competing interests. | | | | | | | |  |
| This statement is required for submission | | | | | | | |  |
| and **will appear in the published article** if | | | | | | | |  |
| the submission is accepted. Please make | | | | | | | |  |
| sure it is accurate and that any funding | | | | | | | |  |
| sources listed in your Funding Information | | | | | | | |  |
| later in the submission form are also | | | | | | | |  |
|  |  |  | |  |  |  |  |  |

*Powered by Editorial Manager® and ProduXion Manager® from Aries Systems Corporation*

declared in your Financial Disclosure

statement.

View published research articles from

[*PLOS ONE*](http://journals.plos.org/plosone/) for specific examples.

**NO authors have competing interests**

Enter: *The authors have declared that no*

*competing interests exist.*

**Authors with competing interests**

Enter competing interest details beginning

with this statement:

*I have read the journal's policy and the*

*authors of this manuscript have the following*

*competing interests: [insert competing*

*interests here]*

* typeset

| **Ethics Statement** | N/A |
| --- | --- |

Enter an ethics statement for this

submission. This statement is required if

the study involved:

- Human participants
- Human specimens or tissue
- Vertebrate animals or cephalopods
- Vertebrate embryos or tissues
- Field research

Write "N/A" if the submission does not

require an ethics statement.

General guidance is provided below.

Consult the [submission guidelines](http://journals.plos.org/plosone/s/submission-guidelines#loc-guidelines-for-specific-study-types) for

detailed instructions. **Make sure that all**

**information entered here is included in the**

**Methods section of the manuscript.**

*Powered by Editorial Manager® and ProduXion Manager® from Aries Systems Corporation*

**Format for specific study types**

**Human Subject Research (involving human**

**participants and/or tissue)**

- Give the name of the institutional review board or ethics committee that approved the study
- Include the approval number and/or a statement indicating approval of this research
- Indicate the form of consent obtained (written/oral) or the reason that consent was not obtained (e.g. the data were analyzed anonymously)

**Animal Research (involving vertebrate**

**animals, embryos or tissues)**

- Provide the name of the Institutional Animal Care and Use Committee (IACUC) or other relevant ethics board that reviewed the study protocol, and indicate whether they approved this research or granted a formal waiver of ethical approval
- Include an approval number if one was obtained
- If the study involved *non-human primates*, add *additional details* about animal welfare and steps taken to ameliorate suffering
- If anesthesia, euthanasia, or any kind of animal sacrifice is part of the study, include briefly which substances and/or methods were applied

**Field Research**

Include the following details if this study

involves the collection of plant, animal, or

other materials from a natural setting:

- Field permit number
- Name of the institution or relevant body that granted permission

| **Data Availability** | No - some restrictions will apply |
| --- | --- |

Authors are required to make all data

underlying the findings described fully

available, without restriction, and from the

time of publication. PLOS allows rare

exceptions to address legal and ethical

concerns. See the [PLOS Data Policy](http://journals.plos.org/plosone/s/data-availability) and

[FAQ](http://journals.plos.org/plosone/s/data-availability#loc-faqs-for-data-policy) for detailed information.

*Powered by Editorial Manager® and ProduXion Manager® from Aries Systems Corporation*

A Data Availability Statement describing

where the data can be found is required at

submission. Your answers to this question

constitute the Data Availability Statement

and **will be published in the article**, if

accepted.

**Important:** Stating ‘data available on request

from the author’ is not sufficient. If your data

are only available upon request, select ‘No’ for

the first question and explain your exceptional

situation in the text box.

Do the authors confirm that all data

underlying the findings described in their

manuscript are fully available without

restriction?

**Describe where the data may be found in**	All relevant data are within the manuscript and its Supporting Information files

**full sentences. If you are copying our**

**sample text, replace any instances of XXX**

**with the appropriate details.**

- If the data are **held or will be held in a public repository**, include URLs, accession numbers or DOIs. If this information will only be available after acceptance, indicate this by ticking the box below. For example: *All XXX files are available from the XXX database (accession number(s) XXX, XXX.*).
- If the data are all contained **within the manuscript and/or Supporting Information files**, enter the following: *All relevant data are within the manuscript and its Supporting Information files.*
- If neither of these applies but you are able to provide **details of access elsewhere**, with or without limitations, please do so. For example:

*Data cannot be shared publicly because of [XXX]. Data are available from the XXX Institutional Data Access / Ethics Committee (contact via XXX) for researchers who meet the criteria for access to confidential data.*

*The data underlying the results*

*presented in the study are available*

*from (include the name of the third party*

Powered by Editorial Manager® and ProduXion Manager® from Aries Systems Corporation

| *and contact information or URL).* |  |
| --- | --- |
| • This text is appropriate if the data are |  |
| owned by a third party and authors do |  |
| not have permission to share the data. |  |
| * typeset |  |
| Additional data availability information: | Tick here if the URLs/accession numbers/DOIs will be available only after acceptance |
|  | of the manuscript for publication so that we can ensure their inclusion before |
|  | publication. |
|  |  |

*Powered by Editorial Manager® and ProduXion Manager® from Aries Systems Corporation*

Manuscript

[Click here to access/download;Manuscript;MS-Gangadhar-25-09-22 - PlosOne.docx](https://www.editorialmanager.com/pone/download.aspx?id=31902043&guid=c59dd00c-14c8-411f-816f-a5f2767b5c62&scheme=1)

1 **Performance of some early-maturing groundnut genotypes and selection of high-yielding genotypes in the**
2 **potato-fallow system**

3

4 Gangadhara K, Ajay BC*, Praveen Kona, Kirti Rani, Narendra Kumar and Bera SK*
5 ICAR-Directorate of Groundnut Research, Junagadh, Gujarat 362001, India
6 *Corresponding author: [berask67@yahoo.co.in,](mailto:berask67@yahoo.co.in) [ajaygpb@yahoo.co.in](mailto:ajaygpb@yahoo.co.in)
7 **Abstract**
8 India is the largest importer of edible oils as it has to meet domestic demand, which surpasses its domestic
9 production. To reduce the import of edible oils, there is a scope for horizontally expanding groundnut production,
10 especially in the potato-paddy rice-fallow system, which requires trait-specific varieties suitable for specific
11 cropping systems. Currently, 99% of oilseeds are cultivated in their traditional areas, while only 1% of cultivation
12 is done in non-traditional areas. To increase the production of oilseeds, both the traditional and non-traditional
13 areas have to be expanded by around 5 million ha according to the strategies of Oilseeds and oil palm by 2020–
14 26 to achieve self-reliance through a campaign called Atmanirbhar Bharat launched by the Government of India
15 (NFSM-Oilseeds and Oil palm). Non-traditional areas are all expanded along river beds, particularly in paddy
16 rice-potato-fallow in the North Eastern States, to utilize residual soil moisture. To assess performance and
17 adaptability, nine interspecific derivatives of groundnut have been evaluated in the potato-fallow system in Deesa
18 in Gujarat and Mohanpura in West Bengal and non-potato and fallow lands in Junagadh during *Kharif* 2020. The
19 combined analysis of variance (ANOVA) showed significant effects of genotype-by-environment (G×E)
20 interaction on pod yield and its component traits. Results of “mean vs. stability” identified the interspecific
21 derivative NRCGCS 446 and the variety TAG 24 as the most stable genotypes with the most useful traits.
22 Genotype GG 7 performed better for pod yield in the Junagadh, whereas NRCGCS 254 performed better in terms
23 of pod yield in Mohanpur. Low heritability estimates and significant G×E interaction found for the days to
24 flowering indicated complex inheritance and influence of environment on their expression. The shelling
25 percentage had a significant negative correlation with days to 50% flowering, days to maturity, SCMR, HPW, and
26 KLWR, suggesting negative associations between maturity, component traits, and seed size realization.
27 **Keywords:** Potato-fallow, Interspecific derivatives, Pod yield, Shelling percentage
28 **Introduction**
29 Though India achieved self-sufficiency in oilseeds during the early 1990s, since 1994, there has been a continuous
30 increase in oil imports. India imports around 15 million tons of edible oils worth approximately Rs 7,300 crore,
31 which accounted for 40% of the agricultural imports bill and 3% of the overall import bill of the country in 2019.
32 With the efforts of the Technology Mission on Oilseeds (TMO), oilseed production increased from 10.8 m.t. in
33 19 m ha of the area during 1985–86 to 25 mt in 27 m ha of the area during 1998-1999. The increase in oilseed
34 production was possible due to the availability of quality seeds of improved cultivars, advanced technology
35 services, and the price support policy. But with the ever-increasing demand for food legumes and oilseeds due to
36 the continuous rise in the population and competition for other remunerative crops, it is necessary to increase
37 oilseed production both horizontally and vertically in the country. Groundnut is a major oilseed crop in India and

1

38 can be grown in three seasons viz., *Kharif*, pre-*rabi*, *rabi,* or summer in different parts of India. However, in India,
39 groundnut is mainly cultivated as a *Kharif* crop under rainfed conditions with an average yield of 1635 kg/ha.
40 However, groundnut cultivation carried out in pre-*rabi*, *rabi*, or summer seasons is more remunerative due to
41 higher productivity (1922 kg/ha) under assured irrigation, better crop management, and low environmental
42 pressure by biotic stresses. It is estimated that about 7-10 lakh ha additional areas could be used for groundnut
43 cultivation as a pre-*rabi/rabi*/summer crop either after *Kharif* paddy or pre-*rabi/rabi* potatoes in lands, which are
44 otherwise kept fallow for some reason or other and are usually referred to as non-traditional areas under groundnut
45 cultivation. These areas are mostly located in Eastern and North-Eastern states of India, which account for 6.08
46 lakh ha of the total *Kharif* area (Triennial average area from 2017-18 to 2019-20, DAC 2022) besides potato-
47 fallow in Uttar Pradesh with the triennial average area of 5.65 lakh ha from 2017-18 to 2019-20, DAC 2021.
48 Rice–fallow cropland areas are the regions of paddy rice cultivation during the *Kharif* growing season that were
49 kept fallow in the *rabi* season due to lack of irrigation, late harvesting of high-yielding long–duration rice varieties,
50 moistures stress at sowing time during the *rabi* season, early withdrawal of the monsoon, waterlogging, excessive
51 moistures in November or December, and crop damage caused by stray cattle and blue bulls (Ali and Kumar
52 2009). Rice fallow (~11.7 million ha) is a rice-based production system, mainly concentrated in the eastern states,
53 i.e., Chhattisgarh, Jharkhand, Upper Assam, Bihar, eastern Uttar Pradesh, Odisha, and West Bengal (Pande et al.
54 2012a; Kumar et al. 2018; Kumar et al. 2019).
55 Although rice-fallow areas receive normal to high rainfall during the rice growing (*Kharif*) season, the majority
56 of rainwater is lost due to high runoff and low moisture storage capacity of soils. Soil compaction after puddling
57 rice restricts water infiltration, and the development of deep, wide cracks in soils after the rice harvest induces a
58 fast depletion of stored soil water through evaporation. Soil moisture stress at the time of fallow crop sowing
59 results in low plant stand (Kumar et al. 2018). Even if the crop is established well with the utilization of residual
60 soil moisture, the lack of winter rain during the reproductive stage often leads to the complete failure of crops
61 (Ghosh et al. 2016). One of the major constraints for utilization of these rice fallows is the lack of short duration
62 (80-90 days) varieties, which can fit into the sowing windows of major crops. Interspecific derivatives represent
63 a good source of valuable alleles conferring various economically important traits. A considerable number of
64 interspecific derivatives have been developed and further used in breeding for groundnut improvement at the
65 ICAR-Directorate of Groundnut Research, Junagadh (Bera et al. 2008; Bera et al. 2013; Bera et al. 2014a; Bera
66 et al. 2014b; Bera et al. 2014c; Bera et al. 2015; Bera et al. 2016; Goswami et al. 2013; Jasani et al. 2021; Kamdar
67 et al. 2013; Kamdar et al. 2021). Efforts are being made to identify early-maturing genotypes suitable for the
68 potato-fallow system.
69 **Materials and methods:**
70 **Experimental details:**
71 Nine interspecific derivatives/mutants/breeding lines were selected out of 450 breeding lines based on the number
72 of days to first flower bud initiation of short-day varieties (data not shown). The experiment involving nine
73 breeding lines, along with three short-duration check varieties (Table 1), was conducted at the ICAR-Directorate
74 of Groundnut Research, Junagadh, during *Kharif* crop season (June to October) in 2017, 2018, and 2019 in a
75 medium black calcareous (12% CaCO3), clayey, Ustochrept soil, with the minimum temperature ranging from

2

76 18.2 to 28.1 °C (February to May) and the maximum temperature ranging from 29.5 to 35.6 °C. The experiment
77 was arranged in a randomized complete block design (RCBD) with three replications. Each accession was planted
78 in a single row of 3 m in length, with a spacing of 60 cm between rows × 10 cm between plants. Standard
79 agricultural practices and plant protection measures were adopted for healthy crop production. Data on days to
80 maturity (DM), days to flower bud initiation (DFI), days to 50% flowering (DFF), SPAD chlorophyll meter
81 readings (SCMR), pod yield per plant (PYPP; g), hundred pod weight (HPW; g), shelling percentage (SP; %),
82 kernel length (KL; mm), kernel width (KW; mm), and kernel length/width ratio (KLWR) were recorded from five
83 randomly selected plants of each genotype under each replication. Days to flower bud initiation and days to 50%
84 flowering were recorded as the number of days from planting to first flowering and the date of planting to reach
85 flowering in 50% of the plants of a genotype, respectively. The surrogate trait for water use efficiency, SCMR,
86 was measured 60 days after planting using a Minolta handheld portable SCMR meter (SPAD-502 plus Minolta,
87 Tokyo, Japan). Other data were recorded at harvest.
88 Based on the initial 3-year evaluation of nine breeding lines at the ICAR-DGR Junagadh, two selected breeding
89 lines (NRCGCS 252 and NRCGCS 446) and four check cultivars (TAG 24, TG 37 A, GG 7, and JL 501) were
90 further evaluated at three locations (Deesa and Junagadh in Gujarat and Mohanpur in West Bengal) during summer
91 2020. The experiments were conducted at Deesa and Mohanpur after harvesting potato crops. Approximately
92 71352 and 68611 ha of fallow potato areas are found in Deesa and Mohanpur (Average area between the periods
93 2016 to 17 and 2019 to 20, DAC 2022), respectively, which can be used for the succeeding groundnut crop
94 cultivation with minimum resources since potato is a crop grown in high input conditions, and residual nutrients
95 available in fallow potato fields meet the major nutrient requirements of the succeeding groundnut crop. Deesa (a
96 part of the Banaskantha district in Gujarat) is characterized by ‘Ustalf’ soil type with the minimum temperature
97 within the ranges of 13.4 to 27.2 °C (February to May) and the maximum temperature ranging from 31.6 to 42.2
98 °C. Mohanpur, a part of the West Medinipur district in west Bengal, is characterized by ‘orthid’ soil type covering
99 45% of the area and ‘Udupts’ soil type covering the remaining 55% of the area. Temperatures in this location
100 range from 16.5 to 27.0 °C (February to May) as minimum and 30.0 to 36.8 °C (February to May) as maximum.
101 Experiments were laid out in randomized complete block design (RCBD) with three replications in all three
102 locations. Standard agronomic practices and plant protection measures were adopted for healthy crop production.
103 Data for pod yield and its related traits were recorded in experiments.
104 **Statistical analyses**
105 The correlation, heritability, and variability parameters were estimated according to the methods suggested by
106 Aljibouri et al. (1958), Johnson et al. (1955), and Panse and Sukhatme (1985), respectively. The stability and pod
107 yield of genotypes were estimated over three years using the Additive Main Effects and Multiplicative Interaction
108 (AMMI) analysis, respectively. AMMI stability value (ASV) for each genotype was calculated using the formula
109 suggested by Purchase (1997) with the Agricolae package (de Mendiburu 2017) in R version 4.1.0 (R core Team,
110 2022). Simultaneous selection indices (SSI) for yield and stability were calculated as suggested by Farshadfar et
111 al. (2011) to identify stable and high-yielding genotypes. The “mean versus stability” biplot was created in the R
112 package GGEBiplotGUI (Bernal and Villardon 2016).
113 **Results and discussion**

3

114 **Preliminary yield evaluation in Junagadh**
115 The combined analysis of variance (ANOVA) showed that mean squares from genotype and environment obtained
116 for pod yield per plant and other studied component traits were significant, except for kernel length/width ratio,
117 indicating significant variation present among the genotypes under study, which offers great scope for
118 improvement of traits through breeding line selection. Similar results have also been reported by Singh et al.
119 (2015), Ajay et al. (2017), Chuni Lal et al. (2019), and Kamdar et al. (2020) for pod yield and its related characters.
120 G×E interactions were significant for pod yield, days to flower bud initiation, days to 50% flowering, hundred
121 pod weight, and kernel weight (Table 2), suggesting that genotypes respond differently from season to season,
122 which is in agreement with the results of earlier studies (Akbar et al. 2017).
123 Mean values for pod yield and its component traits (pooled over three years) are presented in Table 3. The pod
124 yield and its component traits varied over the years. Days to first flowering ranged from 22 days for genotype
125 NRCGCS 62 to 25 days for genotype PM 1, with a mean value of 24 days. As breeding was performed to select
126 early-maturing lines, there was only three days difference between the two extreme genotypes. Such a narrow
127 range was also observed for days to 50% flowering and days to maturity, with the former ranging from 25 to 28
128 days. Days to maturity in total ranged from 97 days (for NRCGCS 254 and NRCGCS 404) to 101 days (for
129 NRCGCS 40), with a mean value of 98 days. SCMR ranged from 21.8 (NRCGCS 330) to 31.3 (NRCGCS 254),
130 with a mean value of 28.45. Pod yield per plant ranged from 4.4 g (NRCGCS 62) to 13 g (NRCGCS 254), with a
131 mean value of 7 g. The hundred pod weight ranged from 55 g (NRCGCS 40) to 82 g (NRCGCS 254), with a mean
132 value of 71 g. Shelling percent ranged from 58% (NRCGCS 40) to 71% (Chico), with a mean value of 66%.
133 Kernel length varied between 9 mm (NRCGCS 330) to 13 mm (NRCGCS 446), with a mean value of 11.1 mm,
134 and kernel length to width ratio was within the ranges of 1.5 mm (NRCGCS 40) to 2.08 mm (NRCGCS 446),
135 with a mean value of 1.78 mm.
136 Breeding lines (genotypes) NRCGCS 254 and NCRGCS 446 showed higher pod yield and larger seed size as
137 compared to check (TAG 24) on average over three years. Two breeding lines, NRCGCS 446 (high average pod
138 yield) and NCGCS 404 (moderate pod yield), and the check variety TAG 24, were found to be the most stable
139 genotypes. Three breeding lines (NRCGCS 404, NRCGCS 254, and NRCGCS 330), on par with TAG 24, seemed
140 to be early-maturing genotypes.
141 **Heritability estimation and Correlation analysis**
142 Analysis of genotypic coefficient variance (GCV), phenotypic coefficient variance (PCV), heritability, and
143 genetic advance of different traits are given in (Table 4). The phenotypic coefficient of variance (PCV) showed a
144 slightly higher value than the genotypic coefficient of variance (GCV) for the majority of traits, indicating that
145 the traits under study were less strongly influenced by the environment. Similar results on groundnut were also
146 reported by Savaliya et al. 2009 and Namrata et al. 2016. The highest PCV (27.1%) and GCV (30.61%) values
147 were found for pod yield per plant. Savaliya et al. (2009) and Namrata et al. (2016) also published similar results.
148 The highest GCV and PVC values indicate the presence of high variability for pod yield, whereas moderate GCV
149 (15.39%) and PCV (16.05%) values were recorded for the hundred pod weight. Low GCV (9.53%) and moderate
150 PCV (10.54%) values were achieved for kernel length. Low GCV and PCV values were reported for days to
151 flower bud initiation (1.77% and 3.16%), days to 50% flowering (0.38% and 2.97%), days to maturity (1.10% and

4

152 1.50%), SCMR (8.31% and 8.95%), shelling percent (5.60% and 6.04%), kernel width (6.91% and 7.76%), and
153 kernel length to width ratio (7.95% and 8.87%), respectively, indicating lower variability existing among the
154 genotypes for these traits. Low variability for these traits is also justified by the fact that the genotypes evaluated
155 were all early-maturing genotypes.
156 Heritability is a good indicator of the transmission of traits from parents to progeny. Heritability is classified as
157 low (below 30%), medium (30%-60%), and high (above 60%). Estimates of heritability help plant breeders use
158 diversity in genetic resources to select superior genotypes. Therefore, high heritability estimates help effectively
159 select any particular character. SCMR, pod yield per plant, hundred pod weight, shelling percent, kernel length,
160 kernel width, and kernel length/width ratio had high heritability values. Moderate heritability was observed for
161 days to maturity (55%), whereas days to flower bud initiation and days to 50% flowering showed low heritability;
162 these results are in agreement with the findings published by Chirinos et al. (2011). Low heritability and genetic
163 advance as percent of mean for days to flowering and days to maturity indicated the influence of environmental
164 factors and polygenic inheritance of traits that are responsible for maturity.
165 The genetic advance is a useful indicator of the effective selection process that can be expected as a result of
166 making the selection on the base population. GA and GAM (Johnson et al., 1955) were classified as low (0-10%),
167 moderate (10.1-20%), and high (> 20%). In the present study, the genetic advance was low for all the traits except
168 for 100-kernel weight (21.6%). High genetic advance as percent of mean and also high heritability was recorded
169 for pod yield per plant and 100-kernel weight, indicating the predominance of additive gene action for these traits.
170 This shows that selection will be effective in improving these traits. High heritability with moderate genetic
171 advance as percent mean was recorded for shelling percent, kernel length, kernel width, and kernel length/width
172 ratio. Moderate heritability and low genetic advance were reported for days to flower bud initiation and days to
173 maturity. Moreover, low heritability and influences of environment on days to flowering and days to maturity
174 were also reported by Kunta et al. (2021)
175 Pod yield showed a significant positive correlation with SCMR, but it was negatively correlated with days to
176 maturity. Shelling percentage showed a significant negative correlation with days to 50% flowering, days to
177 maturity, SCMR, HPW, and KLWR, suggesting a negative relationship between maturity duration and realization
178 of seed size in groundnut (Fig 1).
179 **Evaluation of two interspecific derivatives and four varieties in the potato-fallow system**
180 Pod yield and shelling percent of groundnut genotypes varied among locations, indicating possible crossover GE
181 interactions. Differential performance was also observed among genotypes in potato-fallow in Deesa in Gujarat
182 and Mohanpur in West Bengal and non-potato fallow in Junagadh, Gujarat (Fig 2 and 4). Selection of genotype
183 is difficult when the performance of genotypes varies across different locations, and to overcome this problem
184 and thus, select stable high-yielding genotypes, stability models such as AMMI and GGE biplots are being adopted
185 extensively.
186 Environmental influence on the performance of six genotypes across three locations was studied using the AMMI
187 model, which partitions the total variation due to genotype, environment, and interaction effect of genotype and
188 environment (GEI) (Table 5). For pod yield per plant, environment accounted for 44.04% of the total variation
189 (maximum variation), followed by GEI (31.73%) and genotype (7.56%). For shelling percent, Environment, GEI,

5

190 and genotype accounted for 56.96%, 17.08%, and 14.92% of the total variation, respectively. The contribution of
191 E to the total variation was greater than that of G and GEI for pod yield per plant and shelling percent, indicating
192 that the selection of genotypes based on multi-environment trials for indirect selection for target traits is the best
193 strategy compared to selecting for a trait in one environment to obtain selection gains (Bernardo 2014; Agoyi et
194 al. 2017; Chunilal et al. 2021; Ajay et al. 2021).
195 GEI was partitioned by the AMMI model into two principal components (IPCA1 and IPCA2), which together
196 explained 100% of the total GEI interaction variation for pod yield per plant and shelling percent. For pod yield
197 per plant, IPCA1 could explain 73.7% of the GEI variation, while IPCA2 explained 26.3% of the GEI variation.
198 For shelling percent, IPCA1 explained 78.2%, but IPCA2 explained 21.8% of the GEI variation. Estimates of the
199 average stability index (ASI) of 6 breeding lines for pod yield per plant and shelling percent are presented in Table
200 6. The lower the average stability index (ASI), the more stable the genotype. The stability alone may not serve
201 breeding purposes, and instead, the search for genotypes with high yield and stability is required. Hence,
202 simultaneous selection indices for yield and stability (SSI) were calculated. SSI incorporates both mean pod yield
203 and stability in a single criterion. The low value of this parameter shows desirable genotypes with high mean pod
204 yield and stability. Genotype NRCGCS 446 was the most stable genotype with very low ASI values, whereas the
205 check variety TAG 24 achieved the first rank in terms of SSI and was stable, with high yield. Genotype NRCGCS
206 254 was on par with TAG 24 in terms of stability and yield. For shelling percent, the check variety TG 37A ranked
207 first with respect to SSI, and it was stable, with a high shelling percent.
208 The GGE analysis explains the two most important sources of variation, i.e., the genotype main effect (G) and the
209 genotype × environment (GE) interaction effect, and it has been considered an effective method as the first two
210 principal components (PC1 and PC2) of GGE explained 99% of the total variation. The GGE-biplot analysis is
211 perfectly suitable for the analysis of multiple-environment trials, and it is based on the genetic correlation among
212 the test environments (which-won-where pattern); the evaluation of environment was carried out based on
213 discrimination ability and representativeness, whereas the evaluation of genotype was made based on the mean
214 performance and stability of genotypes across various environments. The GGE biplot graphically displays the
215 G+GE of MET data in a way that facilitates visual variety evaluation and mega-environment identification (Yan
216 et al. 2007; Narendra et al. 2019). The which-won-where pattern of MET data is the polygon view whose
217 visualization is helpful in examining the possible existence of different mega-environments (Yan and Rajcan
218 2002; Yan and Tinker 2006). The lines dividing the biplot into sectors represent a set of hypothetical
219 environments. A polygon is drawn by joining all the genotypes which are located away from the biplot origin. A
220 perpendicular line is drawn from the origin of the biplot, extending beyond the polygon, where the biplot is divided
221 into several sectors. The genotypes located at the vertices in each sector were the best performing genotype in
222 different environments among other genotypes in the same sector. If a genotype located on an angular vertex of
223 the polygon falls into one sector with an environment marker (or several markers), the mean performance of this
224 genotype would be the highest in this particular environment. Another important feature of this biplot is that it
225 indicates environmental groupings, which suggests the possible existence of different mega-environments. Fig 3a
226 and 4a represent a which-won-where pattern for six genotypes across three locations for pod yield and shelling
227 percent, respectively. For pod yield, the genotype JL 501 performed better in Deesa, while NRCGCS 446
228 performed better in Mohanpur, West Bengal, and GG 7 performed better in Junagadh. For shelling percent, the

6

229 genotypes TG 37A and TAG 24 performed better in Deesa and Mohanpur, respectively, whereas JL 501
230 performed better in Junagadh. Such differential ranking of genotypes across different environments suggests the
231 possible existence of crossover GEI. Yan and Hunt (2001), Kaya et al. (2006), Tossim et al. (2020), and Hajjarpoo
232 et al. (2021) also reported a similar differential ranking of genotypes across locations/environments.
233 The GGE biplot ranks the genotypes based on their mean performance and stability across environments (Fig. 3b
234 and 4b). The axis of the “average environment coordination (AEC) abscissa” is denoted by a single arrowed line
235 that passes through the biplot origin with an arrow, which points to higher mean values for the genotypes (the best
236 performing genotypes; high-dormancy genotypes). Therefore, the AEC ordinate is perpendicular to the AEC
237 abscissa. The AEC ordinate approximates the genotypes’ contributions to the G×E interaction, indicating that the
238 closer the genotype to the AEC abscissa, the more consistent or stable the genotype in test environments (Yan
239 2001; Yan and Tinker 2006). Genotype NRCGCS 446 was stable, with a high yield, whereas TAG 24 had high
240 shelling percent and TG 37A was stable in terms of shelling percent.
241 **Conclusion:**
242 Evaluation of groundnut genotypes under the potato-fallow system in different states revealed differential
243 responses of genotypes on pod yield and also higher pod yield under the potato-fallow system compared to that
244 under the non-potato fallow system. Therefore, there is great scope for expanding the groundnut area in potato-
245 fallow lands of India with enhanced productivity. The cultivation of groundnut in potato fallow will not only
246 increase the area but boost the productivity in the country in much shorter crop period and investment. The study
247 identified two stable early-maturing high-yielding breeding lines, NRCGCS 254 and NRCGCS 446, which can
248 be released for commercial cultivation and used further as potential donors for breeding improved groundnut
249 varieties in the potato-fallow system.
250 **Satements:**
251 No grant is received for this study
252 **Conflict of interest:**
253 Authors do not have any conflict of interest.
254 **Acknowledgement:**
255 Authors acknowledge the support and fascilities received from the Director, ICAR-DGR, Junagah, Gujarat.
256 **References:**
258 **Kindly incorporate the below current Reference to safeguard the present results in a better ways, please.**

Ali S, Ahmad R, Hassan MF, Ibrar D, Iqbal MS, Naveed MS, Arsalan M, Rehman A, Hussain T (2022). [Groundnut genotypes’ diversity assessment for yield and oil quality traits through multivariate analysis](https://sabraojournal.org/groundnut-genotypes-diversity-assessment-for-yield-and-oil-quality-traits-through-multivariate-analysis/). *SABRAO J. Breed. Genet*. 54(3): 565-573. http://doi.org/10.54910/sabrao2022.54.3.9

Carvalho, M.J., N. Vorasoot, N. Puppala, A. Muitia and S. Jogloy. 2018. Effects of terminal drought on growth, yield and yield components in valencia peanut genotypes. *SABRAO J. Breed. Genet.* 49(3) 270-279.

Girdthai T, Jogloy S, Vorasoot N, Akkasaeng C, Wongkaew S, Patanothai A, Holbrook CC (2012). Inheritance of the physiological traits for drought resistance under terminal drought conditions and genotypic correlations with agronomic traits in peanut. *SABRAO J. Breed. Genet.* 44(2): 240-262.

Junjittakarn J, S. Jogloy, N. Vorasoot and N. Jongrungklang (2016). [Effect of mid-season drought and recovery on physiological traits and root system in peanut genotypes (Arachis hypogaea L.)](http://sabraojournal.org/wp-content/uploads/2018/01/SABRAO-J-Breed-Genet-48-3-318-331-Junjittakarn.pdf). SABRAO J. Breed. Genet. 48(3): 318-331.

Junjittakarn J, S. Jogloy, N. Vorasoot and N. Jongrungklang (2016). [Root responses and relationship to pod yield in difference peanut genotypes (Arachis hypogaea L.) under mid-season drought](http://sabraojournal.org/wp-content/uploads/2018/01/SABRAO-J-Breed-Genet-48-3-332-343-Junjittakarn.pdf). SABRAO J. Breed. Genet. 48(3): 332-343.

Kasno A and Trustinah (2015). [Genotype-environment interaction analysis of peanut in Indonesia](http://sabraojournal.org/wp-content/uploads/2018/01/SABRAO-J-Breed-Genet-474-482-492-Kasno.pdf). SABRAO J. Breed. Genet. 47(4): 482-492.

Koolachart R, Jogloy S, Vorasoot N, Wongkaew S, Holbrook CC, Jongrungklang N, Kesmala T, Suriharn B (2019). [Association of aflatoxin contamination and root traits of peanut genotypes under terminal drought](http://sabraojournal.org/sabrao-j-breed-genet-51-3-234-251-suriharn/). SABRAO J. Breed. Genet. 51(3): 234-251.

Mahakosee S, Jogloy S, Vorasoot N, Suriharn B, Puppala N, Patanothai A (2015). [Genotypic diversity of traits related to nitrogen fixation in Valencia peanut germplasm](http://sabraojournal.org/wp-content/uploads/2018/01/SABRAO-J-Breed-Genet-474-493-501-Mahakosee.pdf). *SABRAO J. Breed. Genet*. 47(4): 493-501.

Singkham N, Jogloy S, Kesmala T, Swatsitang P, Jaisil P, Puppala N, Patanothai A (2011). Combining ability for oleic acid in peanut (*Arachis hypogaea* L.). *SABRAO J. Breed. Genet.* 43(1) 59–72.

259 Agoyi EE, Odong TL, Tumuhairwe JB, Chigeza G, Diers B W, Tukamuhabwa P. Genotype by environment
260 effects on promiscuous nodulation in soybean (Glycine max L. Merrill). Agr Food Sec. 2017;6: 29.
261 Ajay BC, Bera SK, Singh AL, Narendra Kumar, Dagla MC, Gangadhar K, Meena HN, Makwana AD.
262 Identification of stable sources for low phosphorus conditions from groundnut (Arachis hypogaea L.)
263 germplasm accessions using GGE biplot analysis. Indian J. Genet. 2021; 81(2): 300-306 https://DOI:
264 10.31742/IJGPB.81.2.12.

Ajay BC, Meena HN, Singh AL, Bera SK, Dagla MC, Narendra Kumar, Makwana AD. Response of different

263 peanut genotypes to reduced phosphorous Availability. Indian J. Genet. 2017; 77(1): 105-111.
264 Akbar A, Manohar SS, Murali TV, Sadaiah K, Janila P. Efficient Partitioning of Assimilates in Stress-Tolerant
265 Groundnut Genotypes under High-Temperature Stress. Agronomy 2017; 7: 30.
266 Ali M, Kumar S. Major technological advances in pulses-Indian scenario. Milestones in Food Legumes Research,
267 20 p. (Eds. M Ali and S Kumar). Indian Institute of Pulses Research, Kanpur, India. MoA (2009). Report
268 of Expert Group on Pulses. Department of Agriculture and Cooperation, Ministry of Agriculture, Govt.
269 of India, New Delhi. 2009; pp 9-10.
270 Al-Jibouri HA, Miller PA, Robinson HF. Genotypic and environmental variances and covariance’s in upland
271 cotton crosses of inter-specific origin. Agronomy Journal. 1958; 50: 633-636.
272 Bera SK, Hariprasanna K, Vinod Kumar. NRCG CS–148: A New Large Seeded Genotype of Groundnut Indian
273 J. Plant Genet. Resour. 2008, 21(1):71-74.
274 Bera SK, Ajay BC, Singh AL. WRKY and Na+/H+ antiporter genes conferring tolerance to salinity in
275 interspecific derivatives of peanut (Arachis hypogaea L.). Australian Journal of Crop Science, 2013;
276 *7*(8): 1173.
277 Bera SK, Kasundra SV, Kamdar JH, Ajay BC, Lal Chuni, Thirumalasmy PP, Dash P, Maurya AK. Variable
278 response of interspecific breeding lines of groundnut to *Sclerotium rolfsii* infection under field and
279 laboratory conditions Electronic J Plant Breeding 2014a; 5(1): 22-29.
280 Bera SK, JH Kamdar, AK Maurya, P Dash. Molecular diversity and association of simple sequence repeat
281 markers with bud necrosis disease in interspecific breeding lines and cultivars of peanut (*Arachis*
282 *hypogaea* L.) Australian Journal of Crop Sciences 2014b; 8(5): 771-780.
283 Bera SK, Ajay Basapur Chandrashekar, Sneha Patel, et al . Identification of stable sources for surrogate traits in
284 *Arachis glabrata* and marker-trait association for tolerance to water deficit stress Turk J Bot. 2014c;
285 38: 309-324.
286 Bera SK, JH Kamdar, SV Kasundra, PP Thirumalaisamy. Identification of groundnut genotypes and wild species
287 resistant to stem rot using an efficient field screening technique. Electronic Journal of Plant Breeding.
288 2016; 7(1): 61-70.
289 Bera SK, Kamdar JH, Kasundra SV, Ajay BC. A novel QTL governing resistance to stem rot disease caused by
290 Sclerotium rolfsii in peanut. Australasian Plant Pathol., 2016; 45(6): 637-644.
291 Bera SK, Surendra S Manohar, Murali T Variath, Sunil Chaudhari et al. Assessing variability for disease
292 resistance and nutritional quality traits in an interspecific collection of groundnut (*Arachis hypogaea* L.)
293 Plant Breeding 2018; 137 (6): 883-894.
294 Bernal EF, Villardon PG GGEBiplotGUI: Interactive GGE Biplots in R. available from [https://CRAN.R-](https://cran.r-project.org/package=GGEBiplotGUI)
295 [project.org/package=GGEBiplotGUI.](https://cran.r-project.org/package=GGEBiplotGUI) 2016.
296 Bernardo R. Essentials of plant breeding. Woodbury: Stemma Press; ISBN: 978-0-9720724-2-7; 2014.

8

298 Chirinos FV, Isleib TG, Milla-Lewis SR Breeding for early maturity in peanuts (Arachis hypogaea L.) using
299 traditional methods and marker assisted selection (MAS), PhD thesis, North Carolina State University,
300 Raleigh, North Carolina; 2011.
301 Chuni Lal, Ajay BC, Kajal V Rupapara. AMMI and GGE models indicating seasonal variations as major source
302 of variations for nodulation related characters in peanut. Indian J. Genet., 2021; 81(2): 277-288.
303 Chuni Lal, Ajay BC, Chikani BM, Gor HK. AMMI and GGE biplot analysis to evaluate the phenotypic stability
304 of recombinant inbred lines (RILs) of peanut under mid-season water stress conditions. Indian J. Genet.,
305 2019; 79 (2) 420-426.
306 DAC. Directorate of Economics and Statistics (DES), Ministry of Agriculture and Farmers Welfare (MoA&FW),
307 India. (accessed on 1^st^ July 2022); 2021.
308 De Mendiburu F. agricolae: Statistical Procedures for Agricultural Research. Available from [https://cran.r-](https://cran.r-project.org/web/packages/agricolae/index.html)
309 [project.org/web/packages/agricolae/index.html;](https://cran.r-project.org/web/packages/agricolae/index.html) 2017.
310 Farshadfar E, Mahmodi N, Yaghotipoor A. AMMI stability value and simultaneous estimation of yield and yield
311 stability in bread wheat (Triticum aestivum L.). Aust J Crop Sci, 2011; 5: 1837-1844.
312 Goswami BR, JH Kamdar, SK Bera. Molecular diversity and association of simple sequence repeat markers with
313 kernel mass in cultivated groundnut ( *Arachis hypogaea* L) Australian Journal of Crop Sciences. 2013;
314 7(8): 1152-1159.
315 Hajjarpoora A, Kholová J, Pasupuleti J, Soltani A et al. Environmental characterization and yield gap analysis to
316 tackle genotype-by-environment-by-management interactions and map region-specific agronomic and
317 breeding targets in groundnut. Field Crop Research 2021; 267:108160.
318 Jasani MD, JH Kamdar, Satarupa Bera, Gururaj Sunkad, SK Bera. Novel and stable major QTLs conferring
319 resistance to peanut bud necrosis disease and identification of resistant high yielding peanut breeding
320 lines Euphytica*,* 2021; 217:105, https://doi.org/10.1007/s10681-021-02835-7.
321 Johnson HW, Robinson HF, Comstock RE. Estimation of genetic and environmental variability in soybean.
322 Agronomy J. 1955; 47: 314-318.
323 Kamdar JH, Goswami BR, Bera SK. Genetic molecular diversity in interspecific peanut lines differing in
324 temporal resistance to peanut bud necrosis disease. African Journal of Biotechnology 2013; 13(3):385-
325 393.
326 Kamdar JH, Jasani MD, Ajay BC, Bera SK, Georrge JJ. Effect of selection response for yield related traits in early
327 and later generations of groundnut (Arachis hypogaea L.). Crop Breeding and Applied Biotechnology,
328 2020; 20(2): e317320215.
329 Kamdar JH, Jasani MD, Ajay BC, Kirti Rani, et al. Fatty acid desaturase-2 (ahFAD2) mutant alleles in peanut
330 (*Arachis hypogaea* L.) pre-breeding lines: an insight into the source, features, discourse, and selection of
331 novel pre-breeding lines. Genet Resour Crop Evol. 2021; doi.org/10.1007/s10722-020-00999-0.
332 Kaya Y, Aksura, M, Taner S. CGE-Biplot analysis of multienvironment yield trials in bread wheat. Turkish
333 Journal of Agriculture, 2006; 30, 325-337.

9

334 Kumar R, Mishra JS, Hans H. Enhancing productivity of rice–fallows of eastern India through inclusion of pulses
335 and oilseeds. Indian Farming 2018; 68(08): 7-10.
336 Kumar R, Mishra JS, Rao KK, Kumar R, Singh SK, Bhatt BP. Evaluation of crop establishment techniques in
337 ricefallows of Eastern Indo–Gangetic Plains. Paper presented in the National Conference on “Organic
338 Waste Management for Food and Environmental Security” during 8-10th February 2018; Jointly
339 Organized by ICAR–Indian Institute of Soil Science and Bhopal Chapter of Indian Society of Soil
340 Science, Bhopal at Nabibagh, Berasia Road, Bhopal 462 038 (M.P.), p 26; 2018.
341 Kunta S, Agmon S, Chedvat I, et al. Identification of consistent QTL for time to maturation in Virginia-type
342 Peanut (Arachis hypogaea L.). BMC Plant Biol. 2021; 21, 186 https://doi.org/10.1186/s12870-021-
343 02951-5.
344 Namrata M, Sharma H, Ranwah BR, Bisen P. Variability Assessment and Path Coefficient Analysis in Groundnut
345 (Arachis hypogaea L.) genotypes in Sub- Humid Southern Plains of Rajasthan. Trend s in Biosciences
346 2016; 9(11): 642-646.
347 Narendra Kumar, Ajay BC, Dagla MC, Rathanakumar AL et al. Multi-environment evaluation of Spanish bunch
348 groundnut genotypes for fresh seed dormancy. Indian J. Genet. 2019; 79(3): 571-582.
349 Nigam SN, Nageswara Rao RC, Wynne JC et al. Effect and interaction of temperature and photoperiod on growth
350 and partitioning in three groundnut (*Arachis hypogaea* L.) genotypes. Annals of Applied Biology 1994;
351 125:541-552.
352 Pande S, Sharma M, Ghosh R. Role of pulses in sustaining agricultural productivity in the rainfed rice-fallow
353 lands of India in changing climatic scenario. Climate change and food Security in India: Proceeding of
354 National Symposium on Food Security in Context of Changing Climate, 30 Oct.-1 Nov. 2010, pp. 53-
355 70; 2012.
356 Panse VG, Sukhatme PV. Statistical Methods for Agricultural Workers, Indian Council of Agricultural Research,
357 New Delhi; 1985.
358 Purchase, JL. Parametric analysis to describe genotype × environment interaction and yield stability in winter
359 wheat. Ph.D. thesis, Department of Agronomy, Faculty of Agriculture of the University of the Free State,
360 Bloemfontein, South Africa. http://hdl.handle.net/11660/1966;1987.
361 R Development Core Team. R: A Language and Environment for Statistical Computing. R Foundation for
362 Statistical Computing, Vienna; 2022.
363 Rowland DL, Sorensen RB, Butts CL, Faircloth WH. Determination of maturity and degree day indices and their
364 success in predicting peanut maturity. Peanut Sci. 2006;33(2): 125–36. https://doi.org/10.3146/0095-
365 3679(2006)33[12 5:DOMADD]2.0.CO;2.
366 Savaliya JJ, Pansuriya AG, Sodavadiya PR, Leva RL. Evaluation of inter and intraspecific hybrid derivatives of
367 groundnut (Arachis Hypogaea l.) for yield and its components. Legume Res. 2009; 32 (2): 129-132.
368 Singh AL, Vidya Chaudhari, Ajay BC. Screening of groundnut genotypes for phosphorus efficiency under field
369 conditions. Indian J. Genet. 2015; 75(3): 363-371.

10

370 Tossim HA, Nguepjop JR, Diatta C, Sambou A, Seye M et al. Assessment of 16 Peanut (*Arachis hypogaea* L.)
371 CSSLs Derived from an Interspecific Cross for Yield and Yield Component Traits: QTL Validation.
372 Agronomy 2020; 10:583; doi:10.3390/agronomy10040583.
373 Yan W. GGEbiplot: A windows application for graphical analysis of multi-environment trial data and other types
374 of two-way data. Agron J.2001; 93: 1111-1118.
375 Yan W, Hunt LN. Interpretation of Genotype × Environment Interaction for Winter Wheat Yield in Ontario. Crop
376 Sci. 2001; 41: 19-23.
377 Yan W, Rajcan J. Biplot Analysis of Test Sites and Trait Relations of Soybean in Ontario. Crop Sci. 2002; 42:
378 11-20.
379 Yan WK, Kang MS, Ma BL, Woods S, Cornelius PL. GGE Biplot vs. AMMI analysis of genotype-by-
380 environment data. Crop Sci. 2007; 47: 643-655.
381 Yan,W, Tinker NA. Biplot analysis of multi-environment trial data: Principles and applications. Canadian Journal
382 of Plant Science 2006; 86, 623–645. doi:10.4141/P05-169.

383

384

385

386

387

388

389

390

391

392

393

394

395

396

397

398

399

400

401

402 **Table 1: The available information on the pedigree of nine interspecific derivatives and check**
403 **varieties used in the present study**

| **SN** | **Interspecific derivative** | **Habit Group** | **Pedigree** | |
| --- | --- | --- | --- | --- |
|  |  |  |  | |
| 1 | NRCG CS 254 | SB | J 11 × A. stenosperma | |
|  |  |  |  | |
| 2 | NRCG CS 292 | SB | J 11 × Black testa mutant | |
|  |  |  |  |  |
| 3 | NRCG CS 313 | SB | J 11 | × A. duranensis |
|  |  |  |  |  |
| 4 | NRCG CS 330 | SB | J 11 | × Black testa mutant |
|  |  |  |  | |
| 5 | NRCG CS 400 | SB | GG 2 mutant | |
|  |  |  |  | |
| 6 | NRCG CS 404 | SB | GG 2 mutant | |
|  |  |  |  |  |
| 7 | NRCG CS 445 | SB | J 11 | × A. pusilla |
|  |  |  |  |  |
| 8 | NRCG CS 446 | SB | J 11 | × A. pusilla |
|  |  |  |  | |
| 9 | NRCG CS 62 | VB | CT7-1 × SB XI | |
|  |  |  |  | |
| 10 | Chico (ICG 476) | SB | An early-maturing germplasm line from U S A | |
|  |  |  |  | |
| 11 | Pratap Mungphali 1 (PM1) | SB | ICGV 86033 × ICG-2214) | |
|  |  |  |  | |
| 12 | TAG 24 | SB | Selection from TGE 2 × TGE 1 | |
|  |  |  |  |  |

404

405

406

407

408

409

410

411

412

413

414

415

416

417

418

419

420

421 **Table 2: ANOVA for pod yield, SCMR, and maturity characteristics in the study conducted in**
422 **Junagadh in 2017–2019**

| **EFFECT** | **Df** | **DFI** | **DFF** | **DM** | **SCMR** | **PYL** |
| --- | --- | --- | --- | --- | --- | --- |
|  |  |  |  |  |  |  |
| GEN | **Df** | **DFI** | **DFF** | **DM** | **SCMR** | **PYL** |
|  |  |  |  |  |  |  |
| ENV | 11 | 4.97** | 5.5** | 19.44** | 58.4** | 41.12** |
|  |  |  |  |  |  |  |
| REP(ENV) | 2 | 59.53** | 60.73** | 183.26** | 1124.9** | 13.12** |
|  |  |  |  |  |  |  |
| GEN x ENV | 6 | 3.1** | 2.94** | 10.97 | 12.6 | 2.17 |
|  |  |  |  |  |  |  |
| RESIDUAL | 22 | 3.36** | 5.41** | 8.84 | 7.9 | 8.76** |
|  |  |  |  |  |  |  |
| CV | 66 | 0.86 | 0.81 | 6.67 | 5.60 | 3.04 |
|  |  |  |  |  |  |  |
| L.S.D.(0.05) |  | 3.90 | 3.40 | 2.60 | 8.30 | 25.00 |
|  |  |  |  |  |  |  |
| **EFFECT** |  | 0.87 | 0.84 | 2.43 | 2.23 | 1.64 |
|  |  |  |  |  |  |  |
| GEN | **Df** | **HPW** | **SP** | **KL** | **KW** | **KLWR** |
|  |  |  |  |  |  |  |
| ENV | 11 | 1169.6** | 143.6** | 12.3** | 2.12** | 0.21** |
|  |  |  |  |  |  |  |
| REP(ENV) | 2 | 3006.0** | 2221.6** | 635.7** | 218.3** | 0.03 |
|  |  |  |  |  |  |  |
| GEN x ENV | 6 | 110.0** | 25.5 | 0.3 | 0.29 | 0.02 |
|  |  |  |  |  |  |  |
| RESIDUAL | 22 | 93.7** | 20.6 | 1.2** | 0.41 | 0.05 |
|  |  |  |  |  |  |  |
| CV | 66 | 35.40 | 23.30 | 0.60 | 0.26 | 0.04 |
|  |  |  |  |  |  |  |
| L.S.D. (0.05) |  | 8.30 | 6.80 | 7.00 | 8.00 | 11.70 |
|  |  |  |  |  |  |  |
|  |  | 5.59 | 4.23 | 0.73 | 0.47 | 0.20 |
|  |  |  |  |  |  |  |

423 DFI-Days to first flower bud initiation; DFF-Days to 50% flowering; DMT-Days to maturity; HPW-
424 Hundred pod weight (g); KL-Kernel length (mm); KW-Kernel width (mm); KLWR-Kernel length to width
425 ratio; PYPP-Pod yield per plant; SCMR-SPAD chlorophyll meter readings; SP-Shelling percentage

426

427

428

429 **Table 3: Estimation of genetic parameters for pod yield, SCMR, and maturity characteristics in the**
430 **study conducted in Junagadh in 2017–2019**

|  | **DFI** | **DFF** | **DM** | **SCMR** | **PYLP** | **HPW** | **SP** | **KL** | **KW** | **KLWR** |
| --- | --- | --- | --- | --- | --- | --- | --- | --- | --- | --- |
|  |  |  |  |  |  |  |  |  |  |  |
| Min | 22 | 25 | 97 | 21.82 | 4 | 55 | 58 | 9.43 | 5.26 | 1.52 |
|  |  |  |  |  |  |  |  |  |  |  |
| Max | 25 | 28 | 101 | 31.31 | 13 | 87 | 71 | 13.60 | 6.97 | 2.08 |
|  |  |  |  |  |  |  |  |  |  |  |
| Mean | 24 | 26 | 98 | 28.45 | 7 | 71 | 66 | 11.10 | 6.31 | 1.78 |
|  |  |  |  |  |  |  |  |  |  |  |
| VG | 0.18 | 0.01 | 1.17 | 5.60 | 3.60 | 119.50 | 13.66 | 1.22 | 0.19 | 0.02 |
|  |  |  |  |  |  |  |  |  |  |  |
| VGXE | 0.83 | 1.53 | 0.72 | 0.75 | 1.90 | 19.40 | 0.10 | 0.20 | 0.05 | 0.002 |
|  |  |  |  |  |  |  |  |  |  |  |
| VP | 0.55 | 0.61 | 2.16 | 6.48 | 4.59 | 129.80 | 15.90 | 1.37 | 0.24 | 0.02 |
|  |  |  |  |  |  |  |  |  |  |  |
| GCV | 1.77 | 0.38 | 1.10 | 8.31 | 27.10 | 15.39 | 5.60 | 9.53 | 6.91 | 7.95 |
|  |  |  |  |  |  |  |  |  |  |  |
| PCV | 3.16 | 2.97 | 1.50 | 8.95 | 30.61 | 16.05 | 6.04 | 10.54 | 7.76 | 8.87 |
|  |  |  |  |  |  |  |  |  |  |  |
| h2 (bs) | 33 | 2.0 | 55 | 86 | 79 | 92 | 86 | 90 | 81 | 76 |
|  |  |  |  |  |  |  |  |  |  |  |
| GA | 0.5 | 0.03 | 1.6 | 4.5 | 3.5 | 21.6 | 7.1 | 2.2 | 0.8 | 0.2 |
|  |  |  |  |  |  |  |  |  |  |  |
| GAM | 2.1 | 0.1 | 1.7 | 15.9 | 50.0 | 30.3 | 10.7 | 19.4 | 12.8 | 13.5 |
|  |  |  |  |  |  |  |  |  |  |  |

431

432

433

434

435

436

437

438

439

440

441

442 **Table 4: The performance of genotypes in terms of pod yield, SCMR, and maturity characteristics**
443 **in the study conducted in Junagadh in 2017–2019**

| **S** | **GEN** | **DFI** | **DFF** | **DM** | **SCMR** | **PYLP** | **HPW** | **SP (%)** | **KL** | **KW** | **KLWR** |
| --- | --- | --- | --- | --- | --- | --- | --- | --- | --- | --- | --- |
| **N** |  | **(days)** | **(days)** | **(days)** |  | **(g)** | **(g)** |  | **(mm)** | **(mm)** |  |
|  |  |  |  |  |  |  |  |  |  |  |  |
| 1 | NRCG CS 254 | 23 | 26 | 97 | 31.3 | 13 | 82.3 | 68.0 | 11.5 | 6.3 | 1.9 |
|  |  |  |  |  |  |  |  |  |  |  |  |
| 2 | NRCG CS 292 | 24 | 26 | 98 | 27.7 | 7 | 71.6 | 69.3 | 11.1 | 6.2 | 1.8 |
|  |  |  |  |  |  |  |  |  |  |  |  |
| 3 | NRCG CS 313 | 23 | 26 | 98 | 28.5 | 7 | 74.9 | 68.2 | 11.5 | 6.8 | 1.7 |
|  |  |  |  |  |  |  |  |  |  |  |  |
| 4 | NRCG CS 330 | 23 | 26 | 97 | 21.8 | 5 | 58.5 | 66.9 | 9.6 | 6.1 | 1.6 |
|  |  |  |  |  |  |  |  |  |  |  |  |
| 5 | NRCG CS 40 | 23 | 27 | 101 | 28.9 | 6 | 78.2 | 58.2 | 11.7 | 6.3 | 1.9 |
|  |  |  |  |  |  |  |  |  |  |  |  |
| 6 | NRCG CS 404 | 23 | 26 | 97 | 29.2 | 5 | 55.2 | 71.4 | 9.4 | 6.2 | 1.5 |
|  |  |  |  |  |  |  |  |  |  |  |  |
| 7 | NRCG CS 445 | 24 | 27 | 98 | 30.3 | 7 | 78.6 | 64.4 | 12.2 | 7.0 | 1.8 |
|  |  |  |  |  |  |  |  |  |  |  |  |
| 8 | NRCG CS 446 | 23 | 26 | 100 | 30.4 | 8 | 87.4 | 61.1 | 13.6 | 6.7 | 2.1 |
|  |  |  |  |  |  |  |  |  |  |  |  |
| 9 | NRCG CS 62 | 22 | 25 | 98 | 28.5 | 4 | 56.8 | 63.0 | 9.8 | 5.3 | 1.9 |
|  |  |  |  |  |  |  |  |  |  |  |  |
| 10 | Chico | 24 | 27 | 97 | 26.2 | 7 | 62.1 | 70.5 | 10.7 | 5.8 | 1.9 |
|  |  |  |  |  |  |  |  |  |  |  |  |
| 11 | PM 1 | 25 | 28 | 100 | 30.7 | 6 | 65.5 | 64.9 | 11.0 | 6.3 | 1.7 |
|  |  |  |  |  |  |  |  |  |  |  |  |
| 12 | TAG 24 | 24 | 26 | 97 | 27.9 | 7 | 84.7 | 68.9 | 11.1 | 6.9 | 1.6 |
|  |  |  |  |  |  |  |  |  |  |  |  |
|  | LSD (0.05) | 0.9 | 0.8 | 2.4 | 2.2 | 1.6 | 5.6 | 4.2 | 0.7 | 0.5 | 0.2 |
|  |  |  |  |  |  |  |  |  |  |  |  |

444

445

446

447

448

449

450

451

452

453 **Table 5: Analysis of variance for AMMI model for pod yield per plant and shelling percentage of**
454 **groundnut grown under the potato-fallow system**

| Effect | df | Pod yield per plant | % Var | Shelling percentage | %Var |
| --- | --- | --- | --- | --- | --- |
|  |  |  |  |  |  |
| GEN | 5 | 47.78 | 7.56 | 127.8* | 14.92 |
|  |  |  |  |  |  |
| ENV | 2 | 695.8* | 44.04 | 1219.8** | 56.96 |
|  |  |  |  |  |  |
| GEN x ENV | 10 | 100.2** | 31.73 | 73.2* | 17.08 |
|  |  |  |  |  |  |
| REP(ENV) | 3 | 65.93 | 6.26 | 29.8 | 2.09 |
|  |  |  |  |  |  |
| RESIDUAL | 14 | 23.49 | 10.41 | 27.33 | 8.93 |
|  |  |  |  |  |  |
| IPCA1 | 6 | 182.7 | 73.7 | 146.02 | 78.20 |
|  |  |  |  |  |  |
| IPCA2 | 4 | 97.45 | 26.3 | 61.02 | 21.80 |
|  |  |  |  |  |  |

455

456

457

458

459

460

461

462

463

464

465

466

467

468

469

470

471

472 **Table 6: The AMMI stability value (ASV) and simultaneous selection index for yield and stability (SSI)**
473 **of 6 genotypes for pod yield per plant and shelling percentage**

|  |  | **Pod yield per plant** | | |  |  | **Shelling percentage** | | |  |
| --- | --- | --- | --- | --- | --- | --- | --- | --- | --- | --- |
|  |  |  |  |  |  |  |  |  |  |  |
| **Genotype** | **ASI** | **SSI** | **rASI** | **rY** | **means** | **ASI** | **SSI** | **rASI** | **rY** | **means** |
|  |  |  |  |  |  |  |  |  |  |  |
| **GG7** | 1.50 | 8 | 5 | 3 | 18.87 | 1.85 | 10 | 6 | 4 | 51.8 |
|  |  |  |  |  |  |  |  |  |  |  |
| **JL501** | 2.71 | 11 | 6 | 5 | 16.33 | 1.67 | 7 | 5 | 2 | 57.2 |
|  |  |  |  |  |  |  |  |  |  |  |
| **NRCGCS254** | 0.92 | 6 | 4 | 2 | 21.97 | 0.69 | 6 | 1 | 5 | 50.6 |
|  |  |  |  |  |  |  |  |  |  |  |
| **NRCGCS446** | 0.25 | 7 | 1 | 6 | 15.88 | 1.32 | 10 | 4 | 6 | 45.8 |
|  |  |  |  |  |  |  |  |  |  |  |
| **TAG24** | 0.38 | 3 | 2 | 1 | 22.00 | 0.98 | 5 | 2 | 3 | 55.4 |
|  |  |  |  |  |  |  |  |  |  |  |
| **TG37A** | 0.72 | 7 | 3 | 4 | 16.53 | 1.23 | 4 | 3 | 1 | 57.8 |
|  |  |  |  |  |  |  |  |  |  |  |

474

475

476

477

478

479

480

**Table 7: Weather parameters recorded at Junagadh during the *Kharif* season in 2017-2019**

| **Month** | **June** | |  |  | **July** | |  | **August** | |  | **September** | |  |  | **October** | |
| --- | --- | --- | --- | --- | --- | --- | --- | --- | --- | --- | --- | --- | --- | --- | --- | --- |
|  |  |  |  |  |  |  |  |  |  |  |  |  |  |  |  |  |
| **Year** | **2017** | **2018** | **2019** | **2017** |  | **2018** | **2019** | **2017** | **2018** | **2019** | **2017** | **2018** | **2019** | **2017** | **2018** | **2019** |
|  |  |  |  |  |  |  |  |  |  |  |  |  |  |  |  |  |
| Max Temperature (°C) | 37 | 37.1 | 36.6 | 30.9 |  | 30.7 | 33.9 | 31.3 | 30.5 | 30.4 | 32.4 | 32.1 | 31.1 | 36.5 | 37.4 | 33 |
|  |  |  |  |  |  |  |  |  |  |  |  |  |  |  |  |  |
| Min Temperature(°C) | 26.6 | 28.1 | 27.1 | 25.4 |  | 25.8 | 26.5 | 24.6 | 25 | 25.3 | 24.5 | 23.5 | 25.1 | 22.9 | 21.5 | 23 |
|  |  |  |  |  |  |  |  |  |  |  |  |  |  |  |  |  |
| Mean Temperature(°C) | 31.8 | 32.6 | 31.9 | 28.1 |  | 28.3 | 30.2 | 28 | 27.7 | 27.8 | 28.5 | 27.8 | 28.1 | 29.7 | 29.5 | 28.8 |
|  |  |  |  |  |  |  |  |  |  |  |  |  |  |  |  |  |
| Relative humidity (%) | 69 | 65 | 69 | 85 |  | 85 | 75 | 84 | 83 | 90 | 79 | 71 | 88 | 51 | 47 | 65 |
|  |  |  |  |  |  |  |  |  |  |  |  |  |  |  |  |  |
| Wind speed (Km/h) | 23.9 | 12.5 | 9.8 | 25 |  | 9.3 | 10.7 | 25.3 | 9.3 | 7.5 | 27.4 | 5.2 | 4.1 | 20.2 | 2.7 | 3.1 |
|  |  |  |  |  |  |  |  |  |  |  |  |  |  |  |  |  |
| Evaporation (mm) | 6.8 | 8.2 | 6.8 | 2.4 |  | 2.9 | 4.9 | 3 | 2.9 | 2.5 | 3.9 | 4.3 | 1.5 | 5.9 | 5.5 | 3.6 |
|  |  |  |  |  |  |  |  |  |  |  |  |  |  |  |  |  |
| Total rainfall (mm) | 147.8 | 7.4 | 138 | 330.5 |  | 641.9 | 228 | 282.6 | 88.6 | 393 | 43.5 | 51.5 | 678 | 0 | 0 | 42.2 |
|  |  |  |  |  |  |  |  |  |  |  |  |  |  |  |  |  |
| Total rainy days | 10 | 1 | 7 | 18 |  | 14 | 10 | 10 | 5 | 13 | 4 | 0 | 21 | 0 | 0 | 3 |
|  |  |  |  |  |  |  |  |  |  |  |  |  |  |  |  |  |

18

19

20

Figure	[Click here to access/download;Figure;Figure.docx](https://www.editorialmanager.com/pone/download.aspx?id=31902035&guid=95ec978f-1a34-4f9d-9237-daf8bcd75100&scheme=1)

**Fig 1: Correlation plot showing relationships between pod yield and its component traits**

**Fig 2: Radar plot showing the differential performance of genotypes in potato fallow and non-potato fallow areas**

**Fig 3: GGE biplot of pod yield per plant showing the “which-won-where” pattern and mean vs. stability, indicating the rankings of genotypes (1 = GG7, 2 = JL 501, 3 = NRCGCS 446, 4 = NRCGCS 254, 5 = TG 37A, and 6 = TAG 24)**

**Fig 4: GGE biplot of shelling percentage showing the “which-won-where” pattern and mean vs. stability, indicating the rankings of genotypes (1 = GG7, 2 = JL 501, 3 = NRCGCS 446, 4 = NRCGCS 254, 5 = TG 37A, and 6 = TAG 24)**
