# Supplementary material for: Performance of some early-maturing groundnut (Arachis hypogaea L.) genotypes and selection of high-yielding genotypes in the potato-fallow system
Source: PLoS One. 2023 Apr 25;18(4):e0282438. doi: 10.1371/journal.pone.0282438 (PMC10128977; doi:10.1371/journal.pone.0282438)
Supplement: Attachment — Submitted filename: Reply to comments.docx [file pone.0282438.s003.docx]

Reply to the Reviewers Comments

We are very much thankful and appreciate the Editor and Reviewers for reviewing the manuscript “PONE-D-22-26579” entitled	“*Performance of some early-maturing groundnut genotypes and selection of high-yielding genotypes in the potato-fallow system*”. Your comments were helpful to improve the quality of the manuscript. We have incorporated all the suggested corrections in the revised manuscript. Point-wise response to the comments and suggestions of Editor/Reviewers are placed below:

The revisions in the revised manuscript have been marked with track change mode for easy reference by the Reviewers. Further, we are also submitting a clean (unmarked) version of the revised manuscript for easy reading and review by Reviewers.

We have thoroughly proof-read the revised manuscript (unmarked version) and removed all typo and grammatical errors.

**Reply to the Editor Comments:**

1. Hypothesis of the study are not clear.

Reply: I thank editor for his valuable comments. Hypothesis has been modified to reflect the aim of this study. Kindly see lines 60 and 61 in clean version of manuscript.

2. Manuscript is not written technically sound, and the data do not fully support the conclusions

Reply: As suggested by the reviewer manuscript has been edited to make it technically sound. Conclusions have been modified to suit the results of this study

**Reply to Journal requirements**

1. **Query:** Please ensure that your manuscript meets PLOS ONE's style requirements, including those for file naming.

**Reply:** Manuscript has been modified as per journal format

2. **Query:** Financial disclosure

**Reply**: No grant is received for this study

3. **Query**: We note that you have stated that you will provide repository information for your data at acceptance. Should your manuscript be accepted for publication, we will hold it until you provide the relevant accession numbers or DOIs necessary to access your data. If you wish to make changes to your Data Availability statement, please describe these changes in your cover letter and we will update your Data Availability statement to reflect the information you provide.

**Reply:** All the data have been presented in the manuscript.

4. **Query**: We note that you have included the phrase “data not shown” in your manuscript. Unfortunately, this does not meet our data sharing requirements. PLOS does not permit references to inaccessible data. We require that authors provide all relevant data within the paper, Supporting Information files, or in an acceptable, public repository. Please add a citation to support this phrase or upload the data that corresponds with these findings to a stable repository (such as Figshare or Dryad) and provide and URLs, DOIs, or accession numbers that may be used to access these data. Or, if the data are not a core part of the research being presented in your study, we ask that you remove the phrase that refers to these data.

**Reply:** Agree with the comments and necessary citations have been provided in the manuscript.

5. **Query**: We note you have included a table to which you do not refer in the text of your manuscript. Please ensure that you refer to Table 7 in your text; if accepted, production will need this reference to link the reader to the Table.

**Reply**: I thank editor for valuable suggestions. Table 7 with weather data has been renumbered as Table 2 and suitably cited in the text. Citation of table may be found in line 69 of clean manuscript

**Reply to the Reviewers Comments:**

Reviewer #1

Reply: We thank you for reviewing the manuscript. We have checked the manuscript thoroughly and included all your comments and suggestions in the revised manuscript. The revisions in the revised MNS have been marked with track change for easy reviewing. Besides, we are also submitting the unmarked/clean version of the revised manuscript for easy reading and review.

Highlights:

1. Write the Scientific name

Reply: Thank you for the observations. We do agree with yoru comments and scientific name of crop included in the title

ABSTRACT:

2. Abstract written poorly, seems introduction Suggested to reduced abstract.

Reply: Thank you for the observations. We really apolgies for the same. Abstract has been reduced from 323 words to 174 words. Kindly see lines 8 to 19 in track change mode and in revised version of manuscript

Introduction

3. Introduction is not written well, needs some more data with more references Kindly incorporate the below latest References to better safeguard the present results, please.

Reply: Thank you very much for the nice suggestion. We have restrurued the introduction by incorporating additional data with references. Same highlighted in track change. Kindly see lines 39 to 44 in track mode change and also in clean version of the manuscript.

4. Give references ???

Replay: Thank you for the observations. Reviewer has rightly pointed the missing reference and the same has been included. Kindly see lines 25 in clean version of the manuscript.

5. Give references ??

Reply: suggested correction has been incorporated. Kindly see line 26 in clean version of the manuscript.

6. ??????

Reply: Apologies for misinterpretation, words have been reframed. Kindly see lines 34 in clean version of the manuscript.

7. ????

Reply: Apologies for misinterpretation, units (lakh ha) have been changed to million ha. Kindly see lines 36 in clean version of the manuscript.

8. What reason ??.

Reply: Thank you very much suggested correction done in revised manuscript. Same highlighted in track change. Kindly see lines 38 to 45 in clean version of the manuscript.

9. ?????????????? please mention clearly.

Reply: Apologies for misinterpretation. Suggested correction have been incorporated. Same highlighted in track change. Kindly see lines 38 to 45 in clean version of the manuscript.

10. Not clear. Also give references

Reply: Suggested corrections have been incorporated. Same highlighted in track changes. Kindly see lines 40 in clean version of manuscript.

11. Not clear.

Reply: Sentences were inadvertently used and were not required for manuscript. Hence, they were deleted.

12. Not a proper way to cite references

Reply: Agree with the reviewer and suggested corrections have been incorporated. Kindly see lines 59 and 60 in clean version of manuscript

Material and Methods

13. India?.

Reply: I Thank reviewer for good suggestions and they have been incorporated. Kindly see lines 67 in clean version of manuscript

Results

14. Results are well organised.

Reply: Thank you very much for kind appreciation of reiewer

References

15. References are not according to journal format, follow guideline of the journal. Also check all references in the text and reference list carefully some are missing from the list or not cited in the text.

Reply: I do agree with the reviewer suggestions. References have been formatted as per journal format. All the references cited in text have been correctly listed in text now. Kindly see lines 252 to 400 in clean version of manuscript.

Reviewer #2

Reply: We thank you for reviewing the manuscript and appreciating work. We have checked the manuscript thoroughly and included all your comments and suggestions in the revised manuscript. The revisions in the revised MNS have been marked with track change for easy reviewing. Besides, we are also submitting the unmarked/clean version of the revised manuscript for easy reading and review.

Yours sincerely

Corresponding author:

Ajay BC

Senior Scientist (Plant Breeding)

ICAR-Directorate of Groundnut Research, RRS, Ananthapur-515001

Andhra Pradesh, India

Email: [ajaygpb@yahoo.co.in](mailto:ajaygpb@yahoo.co.in)
